# Ferric Carboxymaltose in the Treatment of Iron-Deficiency Anaemia in Paediatric Patients with Anastomotic Ulcers

**DOI:** 10.3390/children9030378

**Published:** 2022-03-09

**Authors:** Chiara Udina, Maria Andrea Lanzetta, Fulvio Celsi, Egidio Barbi, Giulia Gortani, Matteo Bramuzzo, Grazia Di Leo

**Affiliations:** 1 Department of Medicine, Surgery and Medical Sciences, University of Trieste, 34127 Trieste, Italy; chiaraudina@gmail.com; 2 Institute for Maternal and Child Health—IRCCS “Burlo Garofolo”, 34137 Trieste, Italy; maria.a.lanzetta@gmail.com (M.A.L.); fulvio.celsi@burlo.trieste.it (F.C.); giulia.gortani@burlo.trieste.it (G.G.); matteo.bramuzzo@burlo.trieste.it (M.B.); grazia.dileo@burlo.trieste.it (G.D.L.)

**Keywords:** iron supplementation, short bowel syndrome, chronic enteral bleeding

## Abstract

Objectives: The aim of this paper is to describe a case series of paediatric patients affected by anastomotic ulcers (AU), a late complication of bowel resection in infancy, focusing on the treatment of iron-deficiency anaemia (IDA) with ferric carboxymaltose (FC). Methods: Patients with a diagnosis of AU, treated at the Paediatric Department of the Institute for Maternal and Child Health IRCCS “Burlo Garofolo” from February 2012 to December 2020 were included. Haemoglobin (Hb) values, IDA related symptoms, the need for blood transfusions, for oral or intravenous (iv) iron supplementation and for surgical resections were compared before and after treatment with FC. Adverse effects of FC were recorded. Results: Ten patients with an established diagnosis of AU were identified; eight (8 out of 10) received at least one administration of FC. Lower and higher Hb values increased significantly after treatment (4.9 g/dL vs. 8.2 g/dL, *p* = 0.0003; 9.9 g/dL vs. 13.5 g/dL, *p* = 0.0008 respectively), with a significant reduction of the need for blood transfusions (*p* = 0.0051) and for oral and iv iron supplementation. While receiving standard therapies, seven patients (7 out of 8) complained of asthenia; this symptom resolved in all cases after FC administration. Before FC treatment, two patients (2 out of 8) required surgical resection of AU, with a recurrence of anaemia after a few weeks; after at least one FC infusion, no children needed further bowel resection for IDA. FC caused mild asymptomatic hypophosphatemia in one case. Conclusion: FC appears to be effective and safe in the paediatric population for the treatment of IDA resulting from AU.

## 1. What Is Known

Iron-deficiency anaemia is the main presenting symptom of anastomotic ulcers (AU), a late complication of bowel resection in infancy.

Treatment of AU-related anaemia is challenging because of several factors: a high rate of ulcer relapse, chronic faecal blood loss, low oral iron adsorption due to short bowel syndrome, as well as side effects and the poor practicability of intravenous iron supplementation. 

## 2. What Is New

Ferric carboxymaltose is a novel intravenous iron formulation, which allows the administration of high doses of iron in a short amount of time.

Ferric carboxymaltose appears to be effective and safe in the paediatric population for the treatment of iron-deficiency anaemia resulting from anastomotic ulcers. 

## 3. Introduction

Anastomotic ulcers (AU) are a rare and late complication of intestinal resection. The exact incidence of AU in children has yet to be determined, as different case series have reported it to span between 0.3% and 8% of patients [1,2]. The main clinical sign of AU is iron-deficiency anaemia (IDA), which is due to faecal occult blood loss in absence of systemic inflammation and may cause asthenia and growth retardation. Given the subtle and unspecific presentation, the diagnosis of AU is often delayed: in one of the largest paediatric case series available, the mean diagnostic delay was 3.7 years [1]. Current treatments for AU, such as the administration of proton-pump inhibitors (PPIs), empiric antibiotic therapy, treatment with anti-inflammatory agents and, more recently, local treatment with argon-plasma coagulation and platelet-rich fibrin [3], have had mostly unsatisfactory results. Indeed, recurrences are frequent, and many patients undergo repeated endoscopic or surgical treatments. 

As chronic IDA can have serious consequences in children, including diminished cognitive performance and delayed motor and cognitive development [4], normal iron and haemoglobin (Hb) values should be re-established promptly. However, management of IDA in children suffering from AU can be particularly complex. Indeed, intestinal resection may result in malabsorptive syndromes, thus invalidating the use of oral iron. Iron sucrose has been shown to be an effective option in patients with IDA and malabsorptive syndromes resulting from inflammatory bowel disease [5,6]. However, it can only be administered in limited quantities (300 mg over the course of 2 h) to avoid adverse effects such as hypotension, nausea and dizziness [7]. Further challenges to the management of IDA in children affected by AU are posed by the recurrence of ongoing blood loss and the high rate of relapse after surgery. 

Ferric carboxymaltose (FC) is a novel intravenous iron formulation which allows the administration of high doses of iron (up to 1000 mg) in a short amount of time (15–20 min). FC has been proven to be safe and effective in the treatment of paediatric IDA consequent to IBD or refractory to oral iron therapy [8,9]. 

To the best of our knowledge, there have been no studies on the use of FC in the treatment of IDA consequent to AU. The purpose of this study is to report on the use of FC in a cohort of paediatric patients suffering from AU.

## 4. Methods

We performed a single-center retrospective cohort study. Patients aged <18 years with a diagnosis of AU established through standard endoscopic techniques or capsule endoscopy and treated at the Paediatric Department of the Institute for Maternal and Child Health IRCCS “Burlo Garofolo” from February 2012 to December 2020 were considered eligible for the study. 

According to the Italian Law on the General Authorization for the Processing of Personal Data for Scientific Research Purposes (Authorization no. 9/2014), retrospective archive studies use ID codes, preventing the data from being traced back to the person concerned; therefore, they do not require ethical approval. According to the policy of the Institute, informed consent was signed by a parent at disease onset and at each check-up visit, agreeing that “anonymous clinical data may be used for clinical research purposes, epidemiology, study of pathologies and training, to improve knowledge, care, and prevention”.

Clinical data were extracted from electronic databases. For patients who received at least one dose of iv FC, haemoglobin values were recorded at baseline, 1 and 3 months after infusion and subsequently every 3 months. 

To assess the impact of FC treatment on iron-deficient anaemia, we compared lower and higher Hb values, the prevalence of reported IDA-related symptoms, the requirement of blood transfusions, the need for oral or iv iron supplementation and surgical resections before and after treatment. Any adverse effect related to FC administration was recorded.

Categorical variables are presented as absolute numbers and percentages and compared with chi-square tests or Fisher’s exact tests. Continuous variables were tested for normality with Shapiro–Wilk tests, followed by non-parametric Mann–Whitney U tests. They are described using medians and interquartile ranges (IQRs). 

## 5. Results

**Figure 1:**
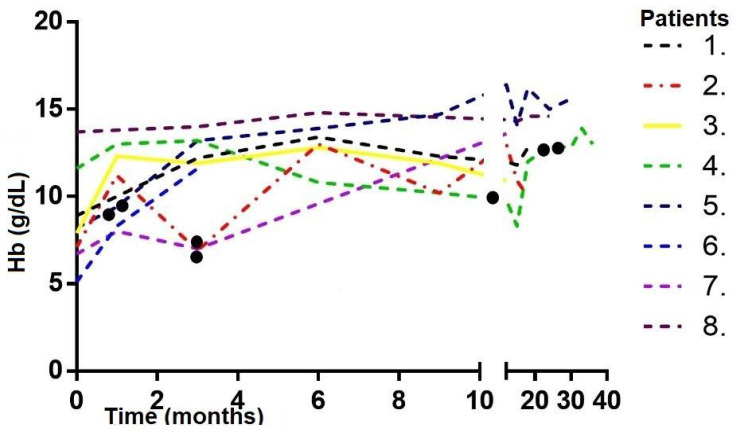
Hb trend after first ferric carboxymaltose infusion (T0) for each patient. Black dots represent further administrations of the drug (patients 2, 4, 5, 7).

**Figure 2:**
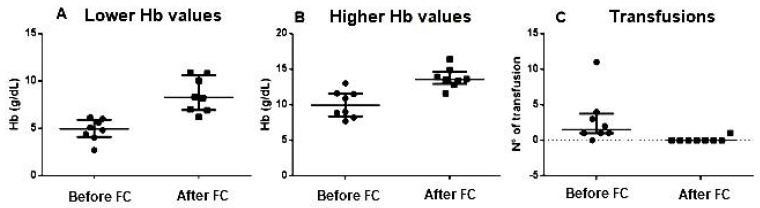
Graphic representation of significant differences between lower values of Hb (**A**), higher values of Hb (**B**) and number of transfusions (**C**) before and after administration of ferric carbox–ymaltose (FC).

Ten patients with an established diagnosis of AU were identified. Demographic and clinical data are reported in Table 1. Disease characteristics are shown in Table 2. All patients had ferritin levels in a range between 1.2 and 4.5 mcg/L at admission. Of our patients, 8 out of 10 received at least one administration of FC during the course of the disease, with a median dosage of 18 mg/Kg (IQR 12.5–20). Among these patients, four (4 of 8) required more than one dose—with a maximum of 4 doses—to achieve and maintain stable Hb values (Figure 1). Median follow-up time was 21.5 months (IQR 9.2–37.3) after the first administration of ferric carboxymaltose. Lower Hb values were significantly higher after treatment than before (8.2 g/dL, IQR 6.9–10.6 vs. 4.9 g/dL, IQR 4.1–5.9, *p* = 0.0003) (Figure 2A). The difference between higher Hb values before (9.9 g/dL, IQR 8.4–11.6) and after FC injection (13.5 g/dL, IQR 12.9–14.6) was also statistically significant (*p* = 0.0008) (Figure 2B). 

The overall improvement in Hb levels resulted in a reduced need for transfusions. Indeed, considering the 8 patients treated with FC, before receiving the first dose all but one (7 out of 8) needed at least one blood transfusion, while only one child required a single blood transfusion after FC injection. The median number of transfusions per patient was significantly greater prior to FC administration (1.5, IQR 1.0–3.7) compared to after FC treatment (*p* = 0.0051) (Figure 2C). 

Before FC treatment, all children received chronic oral iron supplementation and three patients (3 out of 8) underwent multiple iv iron infusions. Conversely, after FC injection only half of the patients (4 out of 8) needed to continue oral supplementation and no further iv iron infusions were necessary. While receiving standard therapies, seven patients out of eight complained of asthenia; this symptom resolved in all cases after ferric carboxymaltose administration. Before FC administration, one fourth of patients (2 out of 8) required surgical resection of AU for IDA, with a recurrence of both anaemia and faecal occult blood loss after 1 month and 2 months respectively. During follow-up after at least one FC infusion, one patient out of eight underwent surgery for intestinal lengthening; no patients needed further bowel resection for IDA. 

FC caused mild asymptomatic hypophosphatemia in one case (patient 5) after the third infusion. 

## 6. Discussion

This study suggests that children with anastomotic ulcers may benefit from FC treatment. 

As described in previous reports [10,11], recurrent ulcer bleeding and the resulting IDA are the major therapeutic challenges of AU. Appropriate management of anaemia is essential in children to prevent serious and long-lasting complications, such as growth retardation and cognitive impairment. To date, all attempted management strategies, both surgical and medical, have largely yielded unsatisfactory results [1]. When specifically considering surgical AU resection, a recurrence rate of up to 100% has been reported in different case series [1,12]. Indeed, also in this case series the two patients who underwent bowel resection in the attempt to treat AU experienced a recurrence of IDA soon after surgery. Remarkably, both were already suffering from short bowel syndrome, as reported for most children with AU. It should be also emphasized that children with short bowel syndrome may present poor absorption of virtually all substances taken orally. Therefore, oral iron supplementation often fails to achieve adequate haemoglobin replacement. Moreover, oral iron frequently results in adverse gastrointestinal effects [13]. For all these reasons, intravenous iron is often the therapy of choice for IDA due to AU. However, its administration by means of repeated and prolonged iv infusion entails the discomfort of multiple iv accesses and the small but reported risk of adverse reactions. 

Formulated as a colloidal stabilized solution, FC is rapidly taken up by macrophages of the reticuloendothelial system and delivered to transferrin, with a small quantity of ionic iron being released into the serum [14]. This allows the administration of high doses of iron in a short amount of time. Moreover, compared to other non-stabilized formulations of iv iron, FC decreases the need for re-injection, while being a safe and effective treatment of paediatric iron-deficiency anaemia [8,9]. 

Prior to treatment with FC, seven of the eight patients in our study were suffering from IDA requiring blood transfusions.

In three children (3 out of 8) a single administration of FC was sufficient to successfully re-establish normal haemoglobin levels in the long term; one of our eight patients received only one infusion before surgical intestinal lengthening that required intraoperative blood transfusion and resulted in a subsequent remission of IDA. 

Significantly, even in the cases requiring multiple injections of the drug (4 of 8), the need for blood transfusions and iv iron was abolished. Moreover, only half of our patients needed to maintain oral iron supplementation, and none continued to complain of asthenia. 

Remarkably, FC resulted in no serious adverse effects, and only caused mild asymptomatic hypophosphatemia in a single patient. 

This study has some limitations, starting with its retrospective design. In addition, an adequate comparison between patients who received treatment with FC and those who did not was not possible, due to the small number of untreated children. Furthermore, AU required treatment with multiple drugs, challenging the rigorous standardization of outcome measures. Nevertheless, we have reported on one of the largest case series of paediatric patients with anastomotic ulcers, with a median follow-up time of 22 months. Finally, while looking mainly at hard outcomes, post-treatment ferritin levels were not available for all patients. In addition, this is the first study analyzing the possible therapeutic role of FC in this setting. 

In conclusion, FC should be considered in the treatment of IDA resulting from AU because of its significant efficacy and safety compared with conventional therapies. Further studies are required to confirm our findings.

## Figures and Tables

**Table 1:** Demographic and clinical characteristics.

Patients	1	2	3	4	5	6	7	8	9	10
Gestational age	35	26	30	25	30	35	33	35	39	37
Gender	F	M	F	M	F	M	F	F	M	F
Underlying condition	Gastroschisis	NEC	NEC	SIP	NEC	Gastroschisis; volvulus	Colonic aganglionosis	IA	IA	IA
Age at surgery (days)	1	30	52	15	6	1	90	1	1	1
Surgical procedure	IR	IR; subtotal colectomy	Ileocolic resection	IR	IR	IR	Colectomy	IR	IR	IR
Anastomosis site	I-C	I-C	I-C	I-I	I-C	I-C	I-R	I-I	I-C	I-I
Presence of ICV	No	No	No	Yes	No	No	No	Yes	No	Yes
Residual SBS	Yes	Yes	Yes	No	Yes	Yes	No	No	Yes	No
Need for PN (months)	22	3	60	-	24	96 (still on PN)	5	10	44	-

IR: ileal resection; IA: ileal atresia; ICV: ileocecal valve; SBS: short bowel syndrome; PN: parenteral nutrition; NEC: necrotizing enterocolitis; SIP: spontaneous intestinal perforation; I-C: ileocolic; I-I: ileoileal; I-R: ileorectal.

**Table 2:** Disease characteristics.

Patients	1	2	3	4	5	6	7	8	9
**Presenting sign/symptom of AU**	IDA	IDA, asthenia	IDA, melena, lipothymia	IDA, asthenia	Rectal bleeding	IDA, asthenia	IDA, asthenia	IDA	IDA, asthenia
**Age at presentation (years)**	14	7	9	5	5	7	8	6	7
**Diagnostic procedure**	IC	IC	CE	CE	CE	CE	IC	CE	IC
**Diagnostic delay (months)**	1	13	13	11	25	10	30	27	1
**Treatment**									
PPIs	Yes →	Yes →	Yes	Yes →	Yes →	Yes *	No	No	Yes
Antibiotics	Yes	Yes →	Yes →	Yes →	Yes →	Yes →	Yes →	Yes →	Yes
Cholestyramine	No	Yes	No	No	No	No	Yes	No	Yes
Mesalamine	Yes →	No	No	No	Yes →	No	Yes →	Yes →	No
Sulfasalazine	Yes →	Yes →	Yes →	Yes →	Yes	Yes →	No	Yes →	Yes
AU resection	No	Yes	No	No	Yes	No	No	No	No
Oral iron	Yes →	Yes →	Yes →	Yes →	Yes	Yes →	Yes	Yes →	Yes
Intravenous iron	No	Yes	No	Yes	Yes	No	No	No	Yes
**FC treatment**	1	2	1	4	3	1	2	1	0
(number of doses)									
**Number of transfusions**									
Before FC	1	4	1	2	11	0	3	1	0
After FC	0	0	0	0	0	1	0	0	-
**Asthenia**									
Before FC	1	1	1	0	1	1	1	1	1
After FC	0	0	0	0	0	0	0	0	-

AU: anastomotic ulcer; IC: ileocolonscopy; PPIs: proton-pump inhibitors; FC: ferric carboxymaltose; IDA: iron deficiency anaemia; CE: capsule endoscopy; → repeated treatment after FC; * treatment only after FC.

## Data Availability

The data presented in this study are available on request from the corresponding author. The data are not publicly available due to privacy reasons.

## Abbreviations

AU: anastomotic ulcer; IDA: iron-deficiency anaemia; PPIs: proton-pump inhibitors; Hb: haemoglobin; FC: ferric carboxymaltose.
